# Molecular Markers and Mechanisms of Influenza A Virus Cross-Species Transmission and New Host Adaptation

**DOI:** 10.3390/v16060883

**Published:** 2024-05-30

**Authors:** Xinyi Guo, Yang Zhou, Huijun Yan, Qing An, Chudan Liang, Linna Liu, Jun Qian

**Affiliations:** 1 School of Public Health (Shenzhen), Shenzhen Campus of Sun Yat-sen University, Shenzhen 518107, China; guoxy89@mail2.sysu.edu.cn; 2 Guangzhou Eighth People’s Hospital, Guangzhou Medical University, Guangzhou 510440, China; 3 Zhongshan School of Medicine, Sun Yat-sen University, Guangzhou 510080, China; yanhj@mail.sysu.edu.cn (H.Y.); liangchd@mail2.sysu.edu.cn (C.L.); 4 College of Wildlife and Protected Area, Northeast Forestry University, Harbin 150040, China; tobyan0515@hotmail.com; 5 Guangdong Provincial Highly Pathogenic Microorganism Science Data Center, Guangzhou 510080, China; 6 Shenzhen Key Laboratory of Pathogenic Microbes and Biosafety, Shenzhen 518107, China

**Keywords:** influenza A virus, cross-species transmission, molecular mechanisms, adaptive mutations, reassortment

## Abstract

Influenza A viruses continue to be a serious health risk to people and result in a large-scale socio-economic loss. Avian influenza viruses typically do not replicate efficiently in mammals, but through the accumulation of mutations or genetic reassortment, they can overcome interspecies barriers, adapt to new hosts, and spread among them. Zoonotic influenza A viruses sporadically infect humans and exhibit limited human-to-human transmission. However, further adaptation of these viruses to humans may result in airborne transmissible viruses with pandemic potential. Therefore, we are beginning to understand genetic changes and mechanisms that may influence interspecific adaptation, cross-species transmission, and the pandemic potential of influenza A viruses. We also discuss the genetic and phenotypic traits associated with the airborne transmission of influenza A viruses in order to provide theoretical guidance for the surveillance of new strains with pandemic potential and the prevention of pandemics.

## 1. Introduction

Influenza viruses are segmented, capsular, negative-sense RNA viruses that are members of the Orthomyxoviridae family. They can be categorized into four groups, A, B, C, and D, according to the antigenic variations in nucleoprotein (NP) and matrix protein (M). Only influenza A viruses are capable of causing viral pandemics [1]. Influenza A viruses are divided into different subtypes based on the antigenicity of their hemagglutinin (HA) and neuraminidase (NA) membrane glycoproteins [2,3]. Wild aquatic birds are natural hosts of influenza A viruses [4]. However, through reassortment or adaptive mutations, some subtypes can overcome the host barrier and spread to poultry and mammals, including people, leading to occasional illnesses, epidemics, and pandemics [5].

Influenza A viruses continue to endanger human health throughout the world and have resulted in significant socio-economic losses. There are approximately 1 billion cases of seasonal influenza that occur annually, with 3–5 million of those cases being severe [6]. An influenza A virus is also a zoonotic pathogen with a multitude of hosts, allowing for interspecies transmission (birds → other mammals/humans), adaptation of new hosts, and the emergence of novel pandemics [7]. Properties that promote interspecies adaptation of viruses include extracellular properties (the stability of virions and the HA protein, virion morphology, balance of HA binding, and NA receptor-destroying activities) and intracellular properties (receptor-binding specificity by the HA protein, HA stability, polymerase efficiency, and interferon antagonism) [8]. Currently, there have been reports of cross-species transmission of avian influenza viruses such as H3N8, H5N6, H5N8, H10N3, and H7N4 [9,10,11,12,13]. The viruses have been observed in mammals by breaking the host barrier through multi-site adaptive changes and reassortment. “Antigenic drift” (accumulation of substitutions in the HA and NA proteins) and “antigenic shift” (recombination of gene segments) are responsible for the phenotypic diversity of influenza A viruses [14,15]. Four global pandemics have been triggered by influenza viruses of the H1, H2, and H3 subtypes in the last 100 years [16]. Unlike seasonal influenza, pandemic viruses result from antigenic switching and are unpredictable [17]. Pre-existing host antibodies offer little protection against these new viruses [18]. Furthermore, animals such as ferrets and pigs, which have both avian and human receptors, play a role as a mixing vessel for the reassortment and generation of the new influenza A virus’s phenotypes [19]. The 2009 pandemic H1N1 (pdmH1N1) was caused by a novel swine-origin recombinant virus, which contained gene segments from three different swine flu lineages [20]. The ability of zoonotic influenza viruses to become airborne should probably be considered an important determinant in crossing the barrier of animal-to-human transmission [21]. Airborne transmission is also the main mode of influenza virus transmission between humans [22]. It has been determined that all influenza viruses responsible for the four recorded pandemics had airborne transmission. This implies that acquiring airborne transmission ability is essential for influenza viruses to adapt to human hosts and have pandemic potential [22]. Consequently, we summarize genetic changes and mechanisms that may influence interspecific adaptation, cross-species transmission, and pandemic potential of influenza A viruses and discuss the phenotypic traits associated with airborne transmission of influenza A viruses. This facilitates the monitoring of the virus and the conduct of risk assessments, as well as the prevention of outbreaks of new pandemic influenza viruses.

## 2. Transmission Modes of the Influenza A Virus

Influenza viruses transmit through multiple modes, including contact (either direct or through a contaminated surface) and inhalation of expelled respiratory droplets or aerosols, the latter two being collectively referred to as airborne [22,23]. In previous studies, droplet diameter was frequently employed as a demarcation between droplet and aerosol transmission, most commonly 5 μm [24]. Droplet transmission (>5 μm) and aerosol transmission (≤5 μm) differ in terms of generation mode, aerodynamics, infectivity, and infection route. Droplets are usually produced by the patient through coughing, sneezing, or speaking, and due to gravity, have a restricted transmission distance and are suspended in the air for a brief period of time [25]. However, viruses are very efficiently spread through aerosols by the patient’s breathing alone [26]. Small aerosols are also more likely to penetrate deep into the alveoli and cause dramatic host responses [26,27]. In addition to the respiratory tract, the conjunctiva and digestive tract are also important routes of aerosol infection [28]. Aerosols may be the main mode of virus transmission in particular scenarios, such as poorly ventilated homes, hospital departments, and wards [29]. It is unclear how much each transmission route contributes to the overall viral propagation capacity, and further research is still required through extensive data collection and modeling. Recently, researchers have questioned the demarcation between droplets and aerosols because it is not based on the well-defined physical properties of droplets or their dynamics in a complex physical environment [30,31]. Viral transmissivity is a continuum that depends on numerous factors (gravitational settling rate, transport, and dispersion in a turbulent air jet, viral load and viral shedding, virus inactivation) that cannot be adequately characterized by a single droplet diameter. Therefore, an airborne transmission mode based on the physical properties of exhaled droplets and their interactions with the environment and human behavior can be used to replace the existing artificial dichotomy [30,32].

Both droplets and aerosols can facilitate efficient host-to-host transmission of influenza viruses. Therefore, it is crucial to comprehend the mechanisms of cross-species and airborne transmission of influenza A viruses and to explore the mutational pathways in the evolution of viruses [21]. Several requirements that we believe are important for influenza A viruses to become airborne are as follows: ① Influenza virions can attach to the appropriate cells in the upper respiratory tract and replicate efficiently. ② Virions are relatively stable and remain infectious while transiting between and within hosts. ③ Virions can be efficiently released outside the cell, leaving the upper respiratory tract as single particles and being expelled in large numbers. The requirements and corresponding mechanisms are summarized in Table 1.

## 3. Molecular Markers and Mechanisms of Influenza A Virus Cross-Species Transmission and New Host Adaptation

### 3.1. Acquisition of HA Human Receptor Binding Preference

The receptor-binding preference of influenza A viruses is critical for successful cross-species transmission of the virus from animal hosts to humans [33]. Studies have demonstrated that avian influenza viruses terminal sialic acid (SA) residues on glycans that are linked to the penultimate galactose through an α-2,3 linkage (SAα-2,3Gal), while human influenza viruses terminal sialic acid residues on glycans that are linked to the penultimate galactose through an α-2,6 linkage (SAα-2,6Gal) [34]. The expression and distribution of SA receptors in tissues may in part contribute to the host range and interspecific adaptation of virus infections [35]. In most avian species, avian-type receptors (SAα-2,3Gal) dominate in tracheal epithelial cells. In contrast, the human upper respiratory tract is rich in SA with α-2,6-linked carbohydrates and thus might be infected by human influenza viruses [36,37]. Both seasonal and pandemic influenza viruses have a high propensity for attaching to human receptors, according to a retrospective analysis by researchers. This preference contributes to virus attachment and efficient replication in the human upper respiratory tract [38]. The human lower respiratory tract contains SAα-2,3Gal, which could explain why avian influenza viruses are able to infect humans and cause a strong host response [39].

The receptor-binding site (RBS) of HA is formed by the 190 helix, the 130 loop, and the 220 loop [40]. RBS has a pocket-like shape with a sialic acid binding site, and amino acid changes in and around it are essential for the specificity of receptor binding and determining the host range of the virus [41]. The molecular mechanisms by which avian-specific HAs gain human receptor-binding avidity vary among different subtypes [42]. E190D and G225D for H1, G226L and G228S for H2 and H3 (HA residues here follow H3 numbering unless otherwise stated) not only confer α-2,6 binding and infection of humans but also successfully spread between human hosts [43,44,45,46]. The main reason for the shift in receptor binding preference is the widening of the binding pocket due to adaptive mutation [47]. It has been suggested that the G225D substitution induced a significant conformational change in the 220-loop, thereby altering the receptor binding specificity [48]. Similarly, the E190D can also expand the binding pocket by changing the conformation of the 220 loop [49]. The Q226L substitution provides a hydrophobic environment and prevents the avian receptor from binding in the trans conformation that is associated with tight binding [50]. The shift of the 220 loop is maximal when both Q226L and G228S substitutions are present [51]. The human H3N8 virus A/Henan/4-10/2022 haemagglutinin has a degenerative codon in position 228, which could be residue G or S [52]. As previously reported for H5N1 and H7N9 viruses, the novel virus could be quickly adapted to human receptor binding by dynamic substitution of key residues of viral proteins [53,54]. In contrast, the adaptive replacements needed for the H5, H6, and H7 subtypes of avian influenza strains to spread in human hosts remain elusive. Observational studies and reverse genetics studies may assist in the discovery of the corresponding genetic changes [55,56]. The two substitutions L129V and A134V, which were identified in a virus isolated from a human case, can change the HA receptor binding preference of H5N1 viruses from SAα-2,3Gal to SAα-2,3Gal and SAα-2,6Gal [55]. H5N1 virus A/duck/Egypt/10185SS/2010 (dkEgy10, clade 2.2.1) contains a Q226L mutation, while N224K, Q226L, N158D, and an L133a deletion were identified in A/chicken/Vietnam/NCVD-093/2008 (ckViet08, clade 7.2) [56]. In conclusion, the structural changes in the RBS of H5N1 viruses mainly focus on alteration of the length of the 130-loop, alteration of a combination of residue positions in the 130-loop and 220-loop, mutations in the 190-helix, and removal of a glycosylation sequon at position 158 [57]. The S123P substitution in HA increases the specificity of the H5N6 virus for human receptor binding. The I151T alteration and residue 129 deletion increase the virus’s ability to bind to human receptors and infect humans [58]. Similar mutations that confer human receptor binding preference on H1, H2, and H3 subtypes were introduced to H6N1 A/Taiwan/2013 viruses. It was found that the G225D mutation completely switches specificity to the human-type receptor [59]. For the H7 subtype, G186V and Q226L mutations may be important for increasing the human receptor binding preference of viruses [60,61]. The hydrophobic milieu surrounding the 220 loop, engendered by the alterations S138A, G186V, T221P, and Q226L, facilitates the attachment of H7N9 viruses to the α-2,6 sialic acid receptor [62]. Several alternative triamino acid mutations (V186G/K-K193T-G228S or V186N-N224K-G228S) have been shown to switch the receptor specificity of H7N9 HA from avian to human [63]. Furthermore, alterations in glycosylation near HA RBS would influence its affinity to receptors [64]. The loss of glycosylation at Asn91 has been demonstrated to affect human receptor binding of H1N1 A/South Carolina/1/18 (SC18) and a single amino acid D225G mutant of SC18 HA (referred to as NY18) by disrupting the network of inter-residue interactions in the RBS of SC18 and NY18 [65]. The N-glycans on HA may also cause steric hindrance near the HA–receptor binding domain, thereby enhancing the affinity to α2,3 sialic acid [66]. The occurrence of adaptive mutations and altered glycosylation in or near the HA receptor-binding site are significant factors that influence viral receptor binding preferences.

Studies of the receptor binding preference of HA in pandemic viruses (those that are able to achieve airborne transmission) revealed that all viruses exhibited high affinity binding to human receptors [67,68,69]. The precise molecular mechanism by which viruses gain airborne transmission remains unclear. It is hypothesized that respiratory droplets or aerosols, which contain few particles, require cells to expose sufficient high-affinity receptors in order to initiate infection and spread between hosts [69]. The relative binding affinity of HA to human receptors correlates with the transmission efficiency of avian influenza viruses in mammalian models such as ferrets. The experimental results of the transmission of influenza A virus H1 [70], H3 [71,72], H5 [73], H7 [74], and H9 [75] subtypes all support this view (Table 2). The acquisition of human receptor binding preference by the Tianjin H9N2 isolate is facilitated by the mutation G226L and seven glycosylation sites (GSs) in the HA protein. Transmission experiments indicated that the virus is efficiently transmitted between guinea pigs and ferrets through direct contact or an airborne route [75]. The two strains of H10N3 virus (A/chicken/Jiangsu/0104/2019 and A/chicken/Jiangsu/0110/2019), which possess the mutation G228S, exhibit dual receptor binding affinity and are highly mammalian-adapted. They can be transmitted between chickens through direct contact and efficiently between guinea pigs through direct contact and respiratory droplets [76] (Table 2). The shift in GS also modifies the transmissibility of the virus. Abdelwhab et al. (2016) discovered that the removal of glycosylation at sites 154 and 236 resulted in a reduction in viral release and a delay in mortality in exposed birds, reducing transmission of the virus from chicken to chicken [77]. The T160A substitution in the A/Vietnam/1203/2004 (H5N1) virus removes the N-linked glycosylation (NLG) sequence at position 158. The removal of GS works synergistically with Q226L or Q226L/G228S mutations to affect the receptor binding preference of the virus, thereby promoting the cross-species transmission of the virus [78,79]. However, possessing a human receptor binding preference is merely the initial stage for the virus to acquire pandemic potential. It needs to be combined with other adaptive traits to gain airborne capacity [76]. The HA G228S mutation increases the virus’s capacity to attach to human receptors. In conjunction with the PB2 E627K alteration, it facilitates the virus’s effective airborne transmission among ferrets [71]. In summary, mutations in RBS and glycosylation modifications around RBS are important factors affecting the receptor-binding specificity of the influenza A virus. Human receptor binding preferences are also important for viruses’ interspecific adaptation and the acquisition of airborne phenotypes.

### 3.2. Changes in HA Stability

A critical stage in the infection phase of the influenza virus is the membrane fusion process mediated by HA. An acidic endosomal environment triggers irreversible conformational changes in the HA protein, leading to viral fusion with the endosomal membrane and injecting nucleic acid into the host cells [85,86]. The optimal pH for membrane fusion varies from host to host [87]. The reasons why HA stability affects the interspecific adaptation and transmission of influenza A viruses are as follows: Firstly, increased HA stability boosts the environmental persistence of influenza viruses and prolongs the half-life of virus transmission between hosts in the external environment [88]. This has been proven by influenza virus transmission tunnel (IVTT) experiments [89]. Secondly, HA with moderate acid stability is indispensable to early conformational changes of HA and prevents virus inactivation before entering mammalian cells [8]. The virus has to pass through the human nasal airway epithelium, which is known to be mildly acidic, before entering the human cells [90]. Finally, HA stability affects membrane fusion sites and triggers varying host responses through distinct pathways, which in turn regulate the infectivity and transmissibility of the virus [90,91]. It is worth noting that the fusion pH of human-adapted subtypes is in general lower than that of their avian counterparts [92]. HA with moderate acid stability is necessary to support airborne transmission (the primary mode of human-to-human transmission) of the virus among ferrets [8].

The stability of HA is largely influenced by electrostatic interactions and van der Waals interactions at domains (RBS, HA1, HA2, and fusion peptide pocket) and subunit interfaces (HA1-HA1 interfaces, HA1-HA2 interfaces) [93,94,95,96]. By regulating these interactions, adaptive mutations affect structural reorganization under viral membrane fusion, thereby changing HA stability [97,98]. Amino acid substitutions, including T642H (HA2 numbering position 64, H1 numbering position HA407), V662H (HA2 numbering position 66, H1 numbering position HA409), T642H, and V662H double mutations, were introduced in the HA1/HA2 interaction region of the A/WSN/33 (H1N1) virus. These substitutions alter the pH of the virus-activated HA by affecting molecular interactions at the HA1 and HA2 interface by a pH of 5.4–5.6, like the wild-type strain, while the V662H and double mutant strains have a lower pH of approximately 5.1–5.3 [99]. Similarly, the HA2-E47K mutation diminishes the membrane fusion pH of the 2009 H1N1 pandemic (pdmH1N1) virus from 5.4 to 5.0. This is due to the formation of a salt bridge between the HA1–E21 residue and the HA2-K47 residue, which stabilizes the HA trimer structure [94]. Several reviews have summarized adaptive mutations that affect the stability of HA acid in influenza A viruses of the H1, H3, H5, and H7 subtypes [96,100]. Key amino acid residues and charged residues located in and around RBS, the HA1-HA1 interface, the HA1–HA2 interface, and the fusion peptide region may play a key role in virus adaptation to a new host and interspecific transmission.

Numerous investigations have confirmed the strong relationship between HA acid stability and the transmission capacity of viruses. For example, in mice and ferrets, the swine influenza virus (pH1N1) A/Tennessee/1–560/2009 was generated via reverse genetics to contain the HA1-Y17H mutation (pH 6.0), which reduces pathogenicity and eliminates airborne transmission capacity. After adaptive culture in ferrets, the loss-of-function virus acquires the HA1-H17Y and HA2-R106K restoration mutations. These two mutations decline the activation pH to 5.3 and restore the virus’s airborne capacity [101]. In another study, the A/Perth/16/2009 (H3N2) after cell culture exhibits mutations at positions 78 and 212 of the HA head (G78D and T212I). The virus’s membrane fusion pH increases from 5.5 (wild type) to 5.8 (mutant). The higher replication titers of mutant viruses in cell culture but lower airborne efficiency in a ferret model suggest that the membrane fusion pH of H3N2 viruses is related to the airborne efficiency of viruses [102]. Hu et al. (2020) compared the transmission capacity of two groups of swine H1N1 influenza viruses (gamma-clade) based on their HA acid stability. The study revealed that strains A/swine/Illinois/2A-1213-G15/2013 (G15) and A/swine/Illinois/2B-0314-P4/2014 (P4), which have moderate stability of HA, gain the ability to be airborne among ferrets. In contrast, viruses with a metastable HA protein (A/swine/Illinois/2E-0113-P19/2013 (P19) pH 5.9) outcompeted and lost the airborne phenotype [103] (Table 3). In conclusion, HA acid stability is an important factor in the airborne transmission of viruses. In addition, the virus-host pH feedback pathway indicates that the virus induces alterations in respiratory tract pH during host infection, and viruses with metastable HA are inactivated extracellularly. Subsequently, the virus increases its infectious dose, and the extracellular pH further decreases, leading to continued virus deactivation. The negative feedback pathway suggests that hosts may select virus strains containing HA proteins with optimal pH stability [104]. It is noteworthy that when an HA mutation associated with increased human receptor binding preference affects HA stability, the virus may require additional compensatory mutations to counteract the decreased stability caused by the mutation [105,106]. This proposes that a precise balance of mutations associated with various functions in HA may be necessary for efficient virus transmission in mammals. 

### 3.3. Functional Balance between the Activities of HA Binding and NA Cleavage

HA and NA of influenza viruses recognize the same host molecule SA and exhibit complementary roles during replication [113]. HA has binding activity and is responsible for binding to the sialic acid to allow virus internalization. NA is a sialidase that mediates the hydrolysis of the link between the SA bound to HA and the adjacent sugar. The cleavage activity of NA prevents the aggregation of nascent virus particles at the surface of the infected cell and allows for viral release [114,115]. In addition to the traditional virion release activity, NA has also been proposed to carry a second SIA-binding site (2SBS) and be involved in the viral entry phase [116]. The 2SBS consists of 370 loops (residues 366–373), 400 loops (residues 399–404), and 430 loops (residues 430–433), which contain residues that interact with SIA (S367, S370, S372, N400, W403, and K432 (N2 numbering)) [117]. It was found that the 2SBS enhances the activity of catalytic sites and affects the HA-NA balance [118]. In addition, HA-NA functional balance affects virus attachment. HA binds to abundant sialylated mucins in the mucus layer, and without NA activity, the viruses may be trapped before they reach the site on the epithelial cell membrane [119,120,121]. NA cleavage activity, which is associated with viral motility in the mucus layer, allows the virion to penetrate the sialylated mucus layer and attach to host cells [122,123]. The sialoglycan repertoire of a host, which varies between species, has a complex composition and distribution of decoy and functional receptors [124]. Influenza A virus’s NA receptor-destroying activity and HA receptor-binding affinity need to be balanced with the host receptor repertoire. When influenza A viruses cross the species barrier, the HA-NA functional balance needs to be restored for optimal viral adaptation [125]. Therefore, the balance between HA binding and NA cleavage activity plays an important role in overcoming host barriers and adapting to new hosts [113]. The optimal functional balance allows viruses to penetrate the sialylated mucus layer and attach to cells, and they can be released from cells after assembly [126].

Any alteration in HA or in NA may cause a modification of the functional HA-NA balance (including mutations affecting HA and NA activity, the addition or removal of glycosylation at key locations, the length of the NA stalk, virus particle morphology, and the NA receptor binding) [118,127,128,129,130]. The HA D222G/E/N substitution increases the binding intensity of influenza A (H1N1) pdm09 virus SAα-2,6, but does not confer the corresponding NA activity, thus disrupting the HA-NA balance. This may be the reason why D222G/E/N viruses are less adaptable to human hosts and cannot transmit efficiently [127]. Mutations affecting the virus particle morphology have been mainly mapped to matrix protein 1 (M1), and these mutations may be related to HA-NA balance and viral transmission ability [128]. Kong et al. found that the M1 D156E substitution increases the proportion of filamentous morphology while decreasing the replicative capacity of the H7N9 A/Anhui/1/2013 (AH/1) virus in cells, thereby abolishing its transmissibility among guinea pigs [131]. The M1 P41A mutation in A/swine/Spain/53207/04 (H1N1) (SPN04) virus reduced the number and length of filamentous morphology and decreased the NA activity of the virus. The enhanced transmissibility of the SPN04 virus may be related to the adjustment of the HA-NA balance [132]. In addition, longer filaments would be able to extend directly through the mucous layer, facilitating airborne transmission of the virus [128]. NLG of HA has been reported to affect its receptor binding and immune response, thereby contributing to the immune escape and virulence of influenza A viruses [66,133]. The NLG of NA impacts its structure, activity, specificity, and thermostability. It was discovered that H5N6 viruses lose a potential n-linked glycosylation site at position 154 and gain an NA mutation at V202I, which affects the balance of HA-NA function, replication, stability, and pathogenicity of the viruses [134]. In addition, it has been found that loss of NLG attenuates viral budding and replication [135,136]. The addition or removal of NLG from the H7 HA head disrupts the HA-NA balance in the viruses, leading to a reduction in viral fitness. This necessitates corresponding changes in N9 NA to restore the balance. Influenza viruses with functional balance can achieve higher transmission efficiencies [129]. Due to the opposite effects of HA and NA in an infection cycle, simultaneous changes in glycosylation patterns should be regarded as a characteristic predicting future pandemic outbreaks of viruses [137]. A shortened NA stalk mainly exists in H5 and H7 subtypes of avian influenza viruses and is also an important factor in the adaptation of waterfowl influenza viruses to poultry [130,138]. The NA stalk length can affect the NA activity of H9N2 G1 lineage virus (8-amino acid-deficient mutants), thereby balancing their HA and NA functions and regulating the entry of viruses into host cells. This effect is species-specific and influences the host range of the virus [139]. It has been demonstrated that the highly pathogenic avian influenza (HPAI) H5N1 viruses with a short stalk exhibit reduced respiratory droplet transmission between ferrets, possibly due to the diminished capacity of viruses with a short-stalk NA to penetrate mucus and deaggregate virions [140]. The 2SBS is an important determinant of the HA-NA-receptor balance. Its effect on HA-NA balance and viral replication depends on the receptor-binding properties of HA [141]. The 2SBS is conserved in most avian virus subtypes but is lost in mammalian variants [118]. Mutations in 2SBS and HA in avian influenza viruses may become human pandemic influenza viruses by adjusting the HA-NA balance [116]. On the one hand, mutations in HA precede and drive mutations in NA that allow optimal viral replication and spread in humans. On the other hand, substitutions in the 2SBS of NA may precede or drive compensatory mutations in HA, decreasing receptor binding to avian-type receptors and increasing human receptor binding affinity [142,143]. In pandemic influenza viruses, the rapid loss of 2SBS binding function may help the virus restore HA-NA stability in a new host [144].

One of the most significant features of airborne influenza A viruses is the high level of individual particle shedding. It is directly affected by the functional balance of HA-NA [22]. Although the mechanism by which the virus acquires an airborne phenotype remains unclear, numerous studies have confirmed the significance of HA-NA functional balance in relation to airborne transmission [69,145,146]. The HA R149K substitution enhances the human receptor binding affinity of triple reassortant swine (TRsw) H1N1 influenza virus A/swine/North Carolina/18161/2002 (NC/02) and its ability to bind to the nasal turbinates of ferrets, thereby promoting contact transmission of the virus. Nevertheless, an airborne phenotype for NC/02 was not established until the introduction of the NA and M segments of the H1N1 pdm09 virus, which yielded NA that matched the highly active HA [145]. Furthermore, the HA-NA balance is crucial for the effective and long-lasting spread of viruses from person to person. The TRsw-like A/swine/Hong Kong/915/04 (sw915) (H1N2) virus exhibits comparable receptor binding specificity and affinity to the pandemic H1N1 virus A/HK/415742/09 (HK415742), with variation only in the NA segment of their internal genes. Introducing the NA segment from HK415742 into sw915 results in balanced HA-NA activity and a developed respiratory droplet transmission of the virus [69]. However, although the A/Iowa/CEID23/2005 (Iowa05) (H1N1) virus has successfully crossed the host barrier to infect humans, the mismatch in HA-NA activity has made it difficult for the virus to spread from person to person and cause a pandemic [146]. In conclusion, the functional balance of HA-NA has been demonstrated to be closely associated with the regulation of host range, cross-species transmission, and airborne transmission of viruses. Further research is warranted to investigate the mechanism by which the functional balance of HA-NA directly impacts the airborne transmission of viruses.

### 3.4. Variations in the Activity of RNA-Dependent RNA Polymerase

RNA-dependent RNA polymerase (RdRp) is a heterotrimeric complex consisting of polymerase basic protein 1 (PB1), polymerase basic protein 2 (PB2), and polymerase acidic protein (PA) [147]. PB1 is the core subunit of RNA synthesis. It facilitates the assembly and synthesis of RdRp and catalyzes RNA polymerization. PB2 binds to PB1 and NP, assisting in the assembly of the viral ribonucleoprotein (vRNP) and capturing the 5′ cap of the nascent host-capped RNA during transcription. It adopts a different conformation to maintain the replication and transcription steps. PA binds to PB1 and plays an important role in endonuclease activity during transcription. All subunits contribute to the formation of entry and exit channels for template RNA, nucleoside triphosphates (NTPs), and products [148,149]. Avian influenza viruses require adaptive mutations or reassortment to enlarge polymerase activity to adapt to human or other mammalian hosts. The PB2 subunit contains well-known mammalian adaptation sites, such as the E627K and D701N mutations, which strengthen viral polymerase activity, replication, pathogenicity, and transmissibility [150,151]. The temperature-dependent growth of the influenza A virus is related to the virus host range [152]. Avian influenza A virus is known to replicate in the intestinal tract of infected birds at a temperature close to 42 °C. The PB2 E627K mutation allows the virus to adapt to the human host and replicate at higher levels in the human respiratory tract (33 °C) [153]. The host factor acidic leucine-rich nuclear phosphoprotein 32 kDa (ANP32) protein displays differential capacity to support viral polymerase activity across different species [154]. Mammals only express the shorter ANP32 protein, which does not efficiently support avian polymerase. However, the presence of PB2 E627K can compensate for the impaired interactions between different host polymerases [155,156]. In addition, it has been demonstrated that the adaptation of the viral polymerase to importin-α plays an important role in interspecies transmission of influenza virus [157]. Although PB2 E627K and D701N mutations caused the same shift to importin-α7 specificity, the adaptation mechanisms are different. The D701N mutation promoted binding of PB2 to importin-α1 and exposure of a nuclear localization signal of PB2, thereby enhancing importin-α mediated nuclear transport in mammalian cells. However, the E627K mutation only enhances the binding of importin-α without affecting the nuclear entry of PB2 [157,158]. Furthermore, changes to the residues in the PA and PB1 subunits have a comparable impact on the virulence, reproduction, and transmission of viruses. For example, by boosting viral polymerase activity, the PA A343S/D347E mutation markedly boosted the virulence of the H5N1 virus in mice and thus elevated the risk of infection in people [159]. Similarly, the PA K356R alteration increases the H9N2 virus’s polymerase activity, which raises viral transcription and export levels in human A549 cells and results in a serious infection in mice [160] (Table 4). Additionally, the PB1 K577E mutation enhances the polymerase activity of the H9N2 virus in human cells and boosts its pathogenicity in mice [161].

Numerous investigations have shown that increased polymerase activity is associated with the acquisition of an airborne phenotype of the virus [162,163]. Effective replication of upper respiratory viruses mediated by polymerase is essential for the airborne transmission of avian influenza viruses among mammals [164]. Experimental data support a strong correlation between the number of influenza viral RNA-containing particles released into the air and airborne transmissibility among ferrets [165]. We speculate that high-titer viral particles are expelled as droplets or aerosols when the donor coughs, sneezes, or even breathes. Once viruses enter the respiratory tract of the recipient host, they replicate efficiently and multiply rapidly, affecting transmissibility and increasing the potential for airborne transmission. Researchers also investigated the impact and corresponding mechanism of polymerase mutation sites on mammalian transmission models [160,162,166]. When replicating within ferrets, recombinant H7N9 viruses with the PB2 E627K and E701N mutations exhibit increased viral polymerase activity and are effectively spread through respiratory droplets [167]. Meng et al. (2022) discovered that four mutations (V100I, N321K, I330V, and A639T) in PA have a synergistic effect on the pathogenicity and airborne transmission of influenza viruses A/swine/Liaoning/265/2017 (LN265) (H1N1). The proposed molecular mechanism suggests that the V100I mutation in PA augments the cleavage activity of the nucleic acid endonuclease. Furthermore, the N321K and I330V mutations develop the binding capacity of vRNA, resulting in the development of the transcriptional efficiency and replication capacity of the virus [168] (Table 4). Similar to Meng et al.’s study, H9N2 avian influenza viruses with the PA K356R mutation demonstrated increased accumulation of PA nuclei, improved viral polymerase activity, and boosted transcriptional replication. The possible molecular mechanisms are as follows: the mutation at site 356, which is in loops 350–355 of the PA-C structural domain, may affect nucleic acid endonuclease activity, protease activity, or interaction with other PA proteins, thereby affecting viral replication and transmission [160]. Less research has been performed recently on the molecular mechanisms of mutation sites in PB1. However, being a fundamental component of RNA synthesis, PB1 is required for viral replication. The wild avian influenza virus A/Mallard/Inner Mongolia/T222/2018 (H3N8) and the virus containing PB1 S524G (rT222-S524G) were saved by reverse genetics. The virus with the PB1 S524G mutation exhibits heightened replication efficiency, increased polymerase activity, and enhanced airborne transmission among ferrets [163]. Moreover, researchers found that the histone protein H1, H2, encoded by HIST1H1C, regulates the ability of influenza viruses to replicate. And specific substitutions on PB2 regulate viral replication by affecting the expression and modification of HIST1H1C [169].

In summary, the improvement of viral polymerase activity facilitates the adaptation of avian influenza viruses to mammalian hosts and enhances their transmission potential, potentially contributing to the acquisition of airborne transmission capability.

**Table 4:** Sites of polymerase mutation that affect the replication and transmission of influenza A viruses.

Host	Viral Subtypes	Subunit (PA/PB1/PB2)	Polymerase Mutation Site	Impact on Viral Replication and Transmissibility	Reference
Mouse	H9N2	PA	K356R	Enhanced viral replication in mice	[160]
Guinea pig	H1N1	PB2	D309N	Virus can be transmitted between guinea pigs by direct contact and has an increased replication capacity	[166]
	H7N9	PB2	V292I K627E	The ability of airborne transmission between guinea pigs is lost	[131]
	H9N2	PB2	R340K A588V	Individual mutations enable guinea pigs to acquire contact transmissibility, and combined mutations facilitate the virus in acquiring airborne transmissibility	[170]
Ferret	H1N1	PA	V100I N321K I330V A639T	The virus is transmitted efficiently between ferrets through respiratory droplets and replicates with higher efficiency	[168]
	H7N1	PB2	T81I	The virus can be transmitted through the air between ferrets when combined with other mutations	[170]
	H3N8	PB1	S524G	The virus is transmitted efficiently between ferrets through respiratory droplets and replicates with higher efficiency	[163]

### 3.5. Reassortment

The genome of the influenza A virus is composed of eight single-stranded, negative-sense RNA segments. Co-infection by different strains in the same host allows for reassortment, resulting in enhanced viral diversity and rapid evolution [171]. It has to be noted that among the four influenza pandemics in the past, three were caused by reassortment [172]. The phenomenon of segment mismatch (including RNA mismatch and protein mismatch between co-infecting viruses) results in the production of progeny viruses with fitness defects [173]. Conversely, reassortant viruses with high levels of genetic compatibility may enhance host adaptability, pathogenicity, and transmissibility and even acquire airborne capability. A reassortant H7N6 virus with internal genes likely derived from H7N9 and H5N6 exhibited comparable binding affinity for both avian-like and human-like receptors and displayed efficient airborne transmission among guinea pigs [174]. The novel reassortant H10N3 virus, whose internal genes are well matched and derived from the H9N2 virus, is transmitted between guinea pigs through direct contact and respiratory droplets [76]. In addition, it has been found that the matching mechanism of HA, NA, and internal gene segments (such as PB2-PB1-PA-NP) is crucial to determining whether influenza A viruses will emerge and successfully transmit among humans [146,175]. The development of reverse genetics techniques has facilitated the assessment of the effects of a single gene on viral phenotypic traits such as virus replication, host range, and transmissibility [176]. The introduction of the HA gene from non-airborne transmissible H7N9 into the genome of the airborne transmissible H9N2 virus decreased acid and heat stability and completely eliminated airborne transmission among chickens. The results demonstrated that the HA gene influenced the airborne phenotypes of H7N9 and H9N2 avian influenza viruses among chickens [177]. The PA gene of the influenza A virus A/swine/Shandong/07/2011 (SD07) (H1N1) was introduced into the avian influenza virus A/Chicken/Shandong/01/2008 (SD01) (H9N2) to obtain the reassortant virus rSD01-PA. rSD01-PA virus lost the ability of airborne transmission among SPF chickens but was still able to infect guinea pigs through direct contact. The findings indicate that the PA gene plays a crucial role in the replication and airborne transmission of the H9N2 avian influenza virus [178]. In conclusion, reassortment is a significant factor in enabling novel host adaptation and cross-species transmission (including airborne transmission, the characteristic of pandemic viruses) of influenza A viruses.

### 3.6. Innate Immune Responses of the Host and Immune Evasion Mechanisms of the Virus

The innate immune system serves as a rapidly responsive first line of defense against influenza A viruses, which helps to restrict viral replication at an early stage and prevent further viral spread [179]. Upon viral infection, pathogen-associated molecular patterns (PAMPs) are recognized by pattern recognition receptors (PRRs) [180]. Activation of these PRRs triggers signaling pathways, leading to the production of interferon (IFN) and proinflammatory cytokines, followed by the expression of interferon-stimulated genes (ISGs) and recruitment of innate immune cells [181]. Many ISG genes have antiviral activity, one of the most potent of these genes is the human myxovirus resistance protein A (MXA). MXA targets the NP of MXA-sensitive viruses and inhibits the transcriptional and replicative functions of viruses. It is an important barrier for cross-species transmission of influenza viruses [182,183]. The tripartite-motif-containing (TRIM) protein TRIM56 has been identified as an intrinsic host restriction factor of influenza A and B viruses. It impedes influenza virus infection by hindering viral RNA synthesis via its C-terminal-tail portion [184]. Moreover, research has demonstrated that eosinophils can initiate a self-preservation response during influenza A virus infection, survive the virus infection, and participate in the antiviral response of the host. The host’s innate immune system restricts the replication and infection of influenza A viruses [185]. Consequently, viruses have to counteract host antiviral activities in order to replicate in host cells. Viral non-structure proteins, nonstructural protein 1 (NS1), and PA-X (a fusion protein), play an important role in the evasion of the host innate immune response by the virus [186]. The NS1 protein counteracts the host antiviral response through a variety of mechanisms, such as inhibiting interferon regulatory factor 3 (IRF3) and nuclear factor kappa-B (NF-κβ) transcription factors, impairing IFN and ISG production [187]. During viral infection, NS1 and PA-X mediate the shutoff of host protein expression and inhibit cellular antiviral responses [188]. Studies have shown that multiple mutations associated with the express ability of host genes have appeared in NS1 and PA-X, and they are most likely related to host adaptation [189]. Therefore, it is necessary to monitor adaptive mutations in the NS1 and PA-X proteins of influenza A viruses.

## 4. Conclusions

Adaptive mutations and reassortment can affect a variety of characteristics of influenza viruses, altering their adaptation, and transmissibility. These characteristics include receptor binding preference, HA stability, HA-NA functional balance, and polymerase activity. Numerous factors influence the choice of adaptation pathways that viruses follow to obtain the desired phenotype. These include the virus’s subtype and strain, compensatory mutations, frequent viral exchange in mixed hosts (like pigs and ferrets), and phenotypic selection for protein function. In summary, a variety of factors influence viral phenotypes, and there exist several routes to phenotype acquisition that consider the features of the virus itself, the interactions between various viruses inside the host, and the type of host.

## Figures and Tables

**Table 1:** Requirements and corresponding mechanisms for cross-species transmission and adaptation of influenza A viruses to new hosts.

Requirements	Corresponding Mechanisms
① Influenza virions can attach to the appropriate cells in the upper respiratory tract and replicate efficiently.	Alteration in HA receptor binding specificity to facilitate viral infection.
	Enhancement of viral polymerase activity to increase the number of viruses in the respiratory tract and efficiently releases them into the air.
② Virions are relatively stable and remain infectious while transiting between and within hosts.	The occurrence of adaptive mutations to improve HA stability.
③ Virions can be efficiently released outside the cell, leaving the upper respiratory tract as single particles and being expelled in large numbers.	Maintenance of the functional balance of HA-NA to produce single-particle viruses
①②③	Reassortment of genes in different segments to help the virus be airborne.

**Table 2:** The HA mutation sites that affect HA receptor binding specificity and transmission of the influenza A virus.

Host	Viral Subtypes	Mutations in HA	Receptor Binding	Impact on Viral Transmissibility	Reference
Guinea pig	H9N2	T187P + M227L	Enhanced affinity for α-2,6 receptors	Effective transmission between guinea pigs by direct contact	[80]
	H1N1	E225G	Reduced binding affinity forα-2,6 receptors.	The ability of droplet transmission between guinea pigs is lost	[81]
Guinea pig and ferret	H9N2	Q226L	Receptor preference changed from α-2,3 to α-2,6 receptors	Effective transmission between guinea pigs through direct contact and airborne routes	[75]
Guinea pig and pig	H3N2	E190D V226I G228S	Receptor preference changed from α-2,3 to α-2,6 receptors	Effective transmission by contact	[82]
Guinea pig	H10N3	G228S	Acquisition of dual receptor affinity for α-2,3 and α-2,6	Effective transmission between guinea pigs by contact and aerosols	[76]
Seal	H3N8	A134T	Developed affinity for α-2,6 receptors	Effective transmission through respiratory droplets (Transmission rates: 100%)	[72]
Dog	H5N6	Q226L Glycosylation deletion at locus 158	Increased affinity for α-2,6 receptors	Crosses the mammalian host barrier and is capable of infecting dogs	[83]
Ferret	H3N8	G228S	Enlarged affinity for α-2,6 receptors	Simultaneous introduction of the HA G228S and PB2 E627K mutation sites can cause viral droplet transmission	[71]
	H5N1	Q226S G228S	Receptor preference changed from α-2,3 to α-2,6 receptors	Effective airborne transmission	[73]
	H9N2	I155T H183N A190V	Improved affinity for α-2,6 receptors	Effective airborne transmission	[84]

Note: Glycine (G), Alanine (A), Valine (V), Leucine (L), Isoleucine (I), Phenylalanine (F), Proline (P), Threonine (T), Tryptophane (W), Serine (S), Tyrosine (Y), Cysteine (C), Methionine (M), Aspartic acid (D), Asparagine (N), Glutamic acid (E), Glutamine (Q), Lysine (K), Arginine (R), Histidine (H).

**Table 3:** The HA mutation sites that affect HA acid stability and transmission of the influenza A virus.

Host	Viral Subtypes	Mutations in HA	Changes in Membrane Fusion pH	Impact on Viral Transmissibility	Reference
Chicken	H7N9	D167N (H7 numbering)	Reduced HA stability	Cannot be transmitted between chickens by air	[107]
	H9N2	K363R (H9 numbering)	Reduced HA stability	Decreased seroconversion rate, Decreased airborne transmission between chickens	[108]
Pig	H1N1	HA1-Y17H HA2-R106K	5.5→6.0	Efficiently transmitted between pigs by contact (Transmission rates: 100%)	[109]
Pig→Ferret (interspecies transmission)	H1N1	HA1-Y17H HA2-R106K	5.5→5.3	Effective airborne transmission between pigs and ferrets (Transmission rates: 100%)	[109]
Mouse and Ferret	H1N1	HA1-H17Y HA2-R106K	6.0→5.3	Virus regains airborne capacity	[101]
Ferret	H3N2	G78D	5.5→5.8	Reduced airborne efficiency	[102]
	H1N1	HA1-N210S HA2-T117N	5.8→5.5 5.9→5.6	Improved airborne transmission between ferrets	[103]
	H3N2	HA1-L194P	<5.5→>5.5	The ability of airborne transmission between ferrets is lost (Transmission rates: 100%→0%)	[110]
	H5N1	H103Y (H5 numbering)	≤5.6→≤5.5	Simultaneous introduction of five mutation sites raises airborne efficiency	[73]
	H10N7	T244I HA2-E74D	5.7→5.2	Transmission between ferrets by aerosols or respiratory droplets	[111]
	H9N2	HA1-Y17H	5.8→5.4	Loss of airborne transmission, only through contact (Less efficient dissemination)	[112]

Note: HA1 and HA2 numbering are the N termini of the HA2 glycoprotein after HA0 cleavage.
